# Adjusting for time of infection or positive test when estimating the risk of a post-infection outcome in an epidemic

**DOI:** 10.1177/09622802221107105

**Published:** 2022-06-12

**Authors:** Shaun R. Seaman, Tommy Nyberg, Christopher E. Overton, David J. Pascall, Anne M. Presanis, Daniela De Angelis

**Affiliations:** 1 MRC Biostatistics Unit, University of Cambridge, East Forvie Building, Forvie Site, Robinson Way, Cambridge, CB2 0SR; 2 Department of Mathematics, University of Manchester, UK; 3 Clinical Data Science Unit, Manchester University NHS Foundation Trust, UK; 4 Joint Universities Pandemic and Epidemiological Research (JUNIPER) consortium; 5 Statistics, Modelling and Economics Department, UKHSA, London, UK

**Keywords:** COVID-19, epidemic phase bias, selection bias

## Abstract

When comparing the risk of a post-infection binary outcome, e.g. hospitalisation, for two variants of an infectious pathogen, it is important to adjust for calendar time of infection. Typically infection time is unknown and positive test time used as a proxy for it. Positive test time may also be used when assessing how risk of the outcome changes over calendar time. We show that if time from infection to positive test is correlated with the outcome, the risk conditional on positive test time is a function of the trajectory of infection incidence. Hence, a risk ratio adjusted for positive test time can be quite different from the risk ratio adjusted for infection time. We propose a simple sensitivity analysis that indicates how risk ratios adjusted for positive test time and infection time may differ. This involves adjusting for a shifted positive test time, shifted to make the difference between it and infection time uncorrelated with the outcome. We illustrate this method by reanalysing published results on the relative risk of hospitalisation following infection with the Alpha versus pre-existing variants of SARS-CoV-2. Results indicate the relative risk adjusted for infection time may be lower than that adjusted for positive test time.

## 1 Introduction

Consider the problem of estimating the distribution of time between a first event (e.g. becoming infected with SARS-CoV-2) and a second event (e.g. testing positive for the virus) in the population of individuals who ultimately experience the second event. We shall call this time the ‘inter-event time’ or ‘delay’. Estimating this distribution may be complicated by the first event time not being observed and/or the available data being right-truncated on the second event time, due to only sampling individuals who experience the second event by a particular calendar time.

It has long been known that the inter-event times in the population of individuals who have experienced the second event before a given calendar time tend to be shorter than in the population of all individuals (who eventually experience the second event) [1, 2, 3]. More precisely, the conditional probability that the inter-event time is less than *l* given that the second event has occurred by a given calendar time is greater than the corresponding unconditional probability (unless all second events have occurred by that time).

It has also been noted that the conditional distribution of inter-event time given the actual calendar time of the second event depends on the marginal distribution of the first event time [4, 5, 6]. In particular, if the first event is generated by a Poisson process whose rate is increasing with calendar time, then the conditional distribution of the inter-event time given the calendar time of the second event is shifted towards zero compared to the unconditional distribution. On the other hand, if the rate is decreasing, the conditional distribution of the inter-event time is shifted away from zero compared to the unconditional distribution. This means that in the context of an infectious disease the time from infection (first event) to positive test (second event) in those who test positive at a given calendar time tends to be shorter than average when the incidence of infection is rising, and longer than average when the incidence is falling.

Now consider a third variable, which is measured at, or after, the time of the second event and is correlated with the inter-event time. Just as the distribution of inter-event time is affected by conditioning on the calendar time of the second event, so might the distribution of this variable. For example, an infected individual’s viral load at time of positive test is a function of time since infection. Rydevik et al. (2016) observed that this relation could be used to estimate an individual’s infection time from that individual’s viral load at the time of testing positive [5]. Hay et al. (2021a) used this same idea to estimate the pattern of incidence of infection in the population from data on the distribution of viral load (measured as the cycle threshold) in a random sample of individuals who tested positive on a given day [6]. If the mean viral load is high, this suggests most of the sampled individuals were infected recently, which is consistent with a rising incidence of infection. Conversely, if the mean viral load is low, this suggests less recent infection, and so a falling incidence. Hay et al. (2021b) investigated using such data to estimate simultaneously the pattern of incidence of infection and the dependence of the viral load on the time since infection [7]. Similar work had previously been done in the field of HIV/AIDS (e.g. [8, 9, 10]).

In the present article we consider the estimation of the distribution of a third variable where this variable is a binary outcome of interest. An association between this binary outcome and the inter-event time could arise due to factors that determine both. We take the first and second events to be infection and positive test, respectively, and the binary outcome to be hospitalisation within 14 days of the positive test, although what follows would apply to any other binary outcome, e.g. death within 28 days of a positive test. Individuals with more severe infections may tend to experience symptom onset sooner after infection — and consequently be tested earlier — than average and also be more likely to become hospitalised. In this situation, the hospitalisation risk (i.e. the proportion ultimately hospitalised) in individuals who test positive before a particular calendar time would be higher than the risk in all individuals who eventually test positive. More importantly for this article, the hospitalisation risk in individuals who test positive *at* a particular calendar time will differ from the risk in all individuals who eventually test positive (unless the incidence of infection is constant over time). If the incidence of infection is rising, the former risk will be higher than the latter; if incidence is falling, it will be lower.

This dependence of the hospitalisation risk on the trajectory of incidence of infection is particularly relevant for any investigation of how the risk is changing over calendar time. Ideally, such an investigation might involve comparing the risks for individuals with different calendar times of infection. If, as is likely, infection times are unknown, it would be natural to instead compare the risks for individuals with different calendar times of positive test. The difficulty with interpreting this latter comparison is that, as noted above, even if the risk does not vary by calendar time of infection, it will depend on calendar time of positive test.

Another situation where one might condition on calendar time of positive test is when comparing the risks associated with two variants of a given pathogen, in this case SARS-CoV-2. Here, controlling for (i.e. conditioning on) time of infection would be important, because the ‘exposure’ (i.e. a binary variable for the variant) and the outcome (hospitalisation) may both depend on calendar time. The exposure would depend on calendar time if the ratio of the incidence rates of infection with the two variants varied over time. That would the case if, for example, one variant emerged earlier but the other variant later became dominant. The hospitalisation outcome would depend on calendar time if measures designed to reduce the need for hospitalisation and/or policies on hospital admission changed over time. Failure to control for infection time when comparing the risks of hospitalisation for the two variants would mean comparing the risk in individuals infected with one variant, whose infection times may have been predominantly when pre-hospital treatments were less effective and/or hospital admission more encouraged, with the risk in individuals infected with the other variant, whose infection times were mostly when pre-hospital treatments were better or hospital admission more restricted. If infection times are unknown, it would be natural to control instead for the time of positive test as a proxy for infection time. The difficulty with this approach is that, even if the hospitalisation risk is the same for both variants and does not depend on time of infection, once we condition on calendar time of positive test a variant that has increasing incidence of infection will appear to have a higher risk than a variant that has a decreasing incidence.

Numerous studies have compared the risks of hospitalisation, intensive care unit admission and/or death in individuals infected with two variants of SARS-Cov-2 (either Alpha versus pre-existing non-Alpha or Delta versus Alpha), adjusting for calendar time of positive test, e.g. [11, 12, 13, 14, 15, 16, 17, 18, 19, 20, 21, 22]. In all these studies, the incidence of one variant has been rising while the other has been falling or has been rising at a slower rate.

In this article, we describe in detail why and how the conditional risk of hospitalisation given time of positive test depends on the trajectory of incidence of infection, even when the conditional risk given time of infection does not. We also propose an easily implemented method that provides an indication of how much an estimate of the risk conditional on the positive test time might differ from the estimate one would have obtained if it had been possible to condition on the infection time. This method involves calculating the risk conditional on a *shifted* positive test time. For each individual who is not ultimately hospitalised, this shifted test time is the same as the actual positive test time, i.e. there is no shift. However, for each individual who is ultimately hospitalised, the shifted time equals the actual positive test time plus the difference between the mean time from infection to positive test in individuals who do not become hospitalised and the mean time in individuals who do become hospitalised. This ensures that the shifted time from infection to positive test is uncorrelated with the hospitalisation outcome. Because this difference between mean times is unknown, our method requires the user to specify a range of plausible values for it.

The structure of the article is as follows. Section 2 defines our notation. Section 3 describes why and how the distribution of the delay conditional on the calendar time of positive test depends on the incidence of infection. Section 4 goes on to explain how this dependence affects the conditional risk of hospitalisation given calendar time of positive test. We introduce our proposed method in Section 5, and its performance is studied in Section 6. Practical application of the method is detailed in Section 7, and Section 8 illustrates its use on COVID-19 data from England. We conclude with a discussion in Section 9.

## 2 Notation

We shall consider the population to be everyone who is at risk of infection from some calendar time zero. Time can be measured discretely or continuously. Suppose for now that all infections result in positive tests. In Section 9 we shall discuss the consequences of violation of this assumption. If an individual has two or more separate episodes of infection, we only consider the first episode.

For each individual in the population, let *I* denote the (calendar) time of infection, and let *L* be the delay (‘*L*’ for ‘lag’) between infection and positive test. Now, *T* = *I* + *L* is the time of positive test. Let *H* equal 1 if the individual is hospitalised within 14 days of positive test, and 0 otherwise. In Section 7 we shall use *V* to denote a binary indicator of which of two variants has infected an individual, and use *U* to denote a vector of variables that are fixed from the time of infection, e.g. age and ethnicity. There we shall be interested in the odds ratio of hospitalisation associated with *V* adjusted for *U* and *I*.

## 3 Delay distribution conditional on test time

In this section and Section 4, we shall assume, for simplicity, that *L* is independent of *I*. Using Bayes’ Rule, *f_L_*(*l* | *T* = *t*), the conditional probability distribution function of the delay given the positive test occurs at time t, can be shown to be related to the unconditional probability distribution function *f_L_*(*l*) by

(1) $$f_{L}\left(l\;|\;T=t\right)=\frac{f_{T,L}\left(t,l\right)}{f_{T}\left(t\right)}=\frac{f_{I,L}\left(t-l,l\right)}{f_{T}\left(t\right)}=f_{L}\left(l\right)\times \frac{f_{I}\left(t-l\right)}{f_{T}\left(t\right)}$$

If the incidence of infection is increasing over the period prior to time *t*, *f_I_*(*t* – *l*) will be a decreasing function of *l*. So, for any *l*_1_ < *l*_2_, we have *f_I_*(*t* – *l*_1_) > *f_I_*(*t* – *l*_2_) and so equation (1) implies

(2) $$\frac{f_{L}\left(l_{1}\;|\;T=t\right)}{f_{L}\left(l_{1}\right)}=\frac{f_{I}\left(t-l_{1}\right)}{f_{T}\left(t\right)}>\frac{f_{I}\left(t-l_{2}\right)}{f_{T}\left(t\right)}=\frac{f_{L}\left(l_{2}\;|\;T=t\right)}{f_{L}\left(l_{2}\right)}.$$

From inequality (2), we have

$$\frac{f_{L}\left(l_{1}\;|\;T=t\right)}{f_{L}\left(l_{2}\;|\;T=t\right)}>\frac{f_{L}\left(l_{1}\right)}{f_{L}\left(l_{2}\right)}.$$

That is, conditioning on *T* = *t* shifts probability mass from larger values of *L* to smaller values. So, if we look only at those individuals whose positive test time is *t*, then small delays will be over-represented and long delays under-represented. This is not surprising, since an individual with positive test time *t* had a short delay if he was infected recently and a long delay if he was infected long ago, and there are more individuals infected recently than individuals infected long ago.

Conversely, if the incidence of infection is decreasing, *f_I_* (*t* – *l*) will be a increasing function of *l*, and so

$$\frac{f_{L}\left(l_{1}\;|\;T=t\right)}{f_{L}\left(l_{2}\;|\;T=t\right)}<\frac{f_{L}\left(l_{1}\right)}{f_{L}\left(l_{2}\right)}.$$

That is, conditioning on *T* = *t* shifts probability mass from smaller values of *L* to larger values: long delays are over-represented and short delays under-represented. In this situation, there are fewer individuals infected recently than individuals infected long ago.

### Example 1

**Figure 1:**
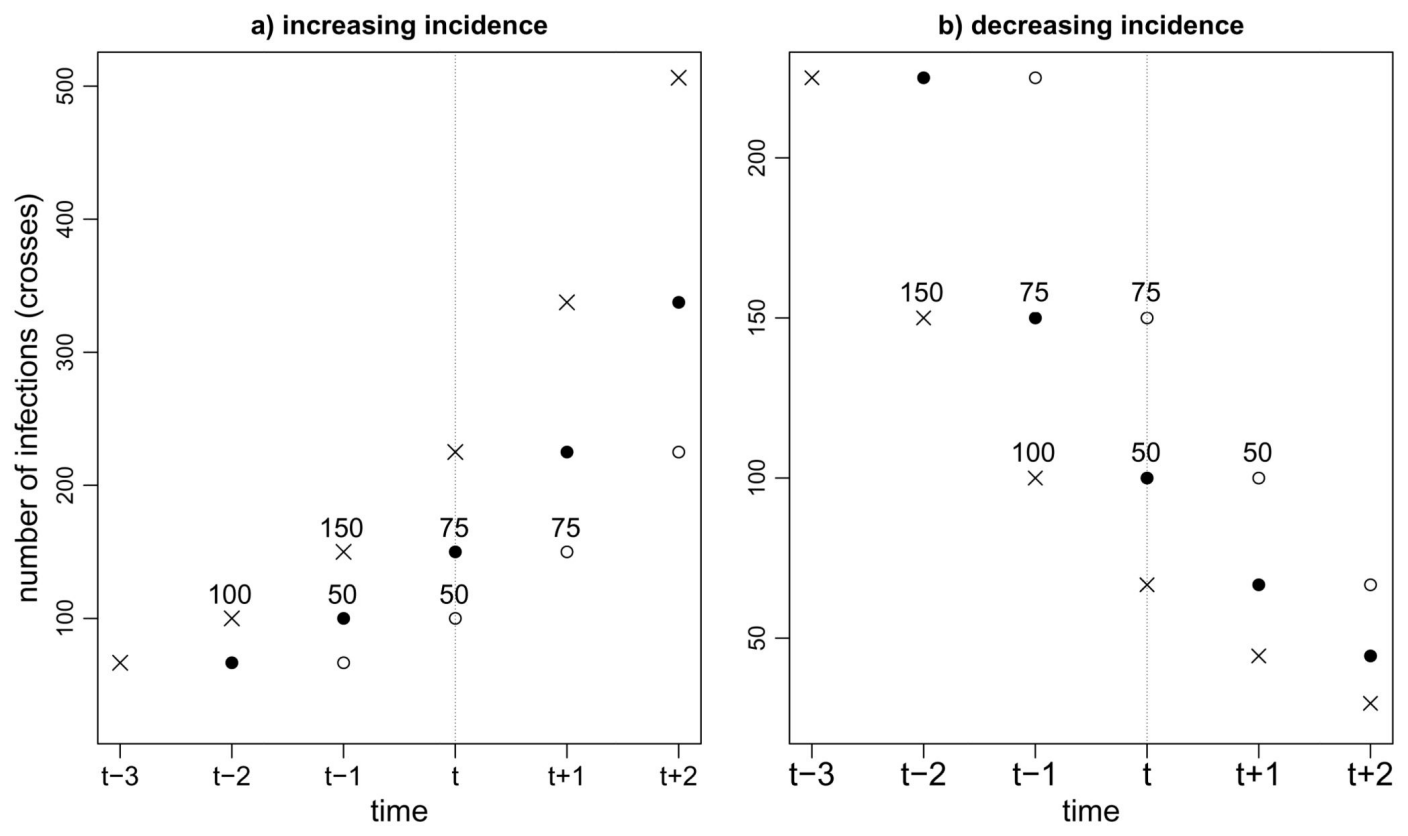
Illustration of Example 1. Crosses show numbers of infections. Black and white circles represent cases with delays of 1 and 2 days, respectively. In left-hand graph, infection incidence is increasing. 100 individuals are infected on day *t* – 2, of whom half (i.e. 50) test positive with a delay of 2 days on day *t*. In addition, 150 individuals are infected on day *t* – 1, of whom half (i.e. 75) test positive with a delay of 1 day on day *t*. So, 75/(50 + 75) = 60% of the cases who test positive on day *t* have a delay of 1 day. In right-hand graph, incidence is decreasing. 150 individuals are infected on day *t* – 2, of whom 75 test positive with a delay of 2 days on day *t*. 100 individuals are infected on day *t* – 1, of whom 50 test positive with a delay of 1 day on day *t*. So, 50/(50 + 75) = 40% of the cases who test positive on day *t* have a delay of 1 day.

Suppose half of infected individuals test positive on the day after they are infected and the other half test positive two days after they are infected. That is, *P*(*L* = 1) = *P*(*L* = 2) = 0.5. Further, suppose that 100 individuals are infected on day *t* – 2 and 150 individuals are infected on day *t* – 1 (so incidence is increasing). Then 125 individuals will test positive on day *t* and, of these, 50 were infected on day *t* – 2 and 75 were infected on day *t* – 1. So, the proportion of these 125 individuals whose delay was one day is 75/125 = 0.6 > 0.5. Conversely, suppose that 150 individuals are infected on day *t* – 2 and 100 individuals are infected on day *t* – 1 (incidence is decreasing). Then 125 individuals will again test positive on day *t*, but the proportion of these whose delay is one day is only 50/125 = 0.4 < 0.5. Figure 1 illustrates this example.

### Example 2

Verity et al.[23] (see also [3]) showed that if infections are generated by a Poisson process with rate at time *t* proportional to exp(*λt*) for some *λ*, and the delay *L* has a gamma distribution with shape *α* and rate *β*, then the conditional distribution of *L* given *T* = *t* is gamma with shape *α* and rate *β* + *λ*. If *λ* > 0, then the incidence is rising (exponentially) and the conditional mean delay, *α*/(*β* + *λ*), is less than the unconditional mean *α*/*β*. If instead *λ* < 0, then the incidence is falling and the conditional mean delay is greater than the unconditional mean. If *λ* = 0, the two gamma distributions are the same.

### Example 3

**Figure 2:**
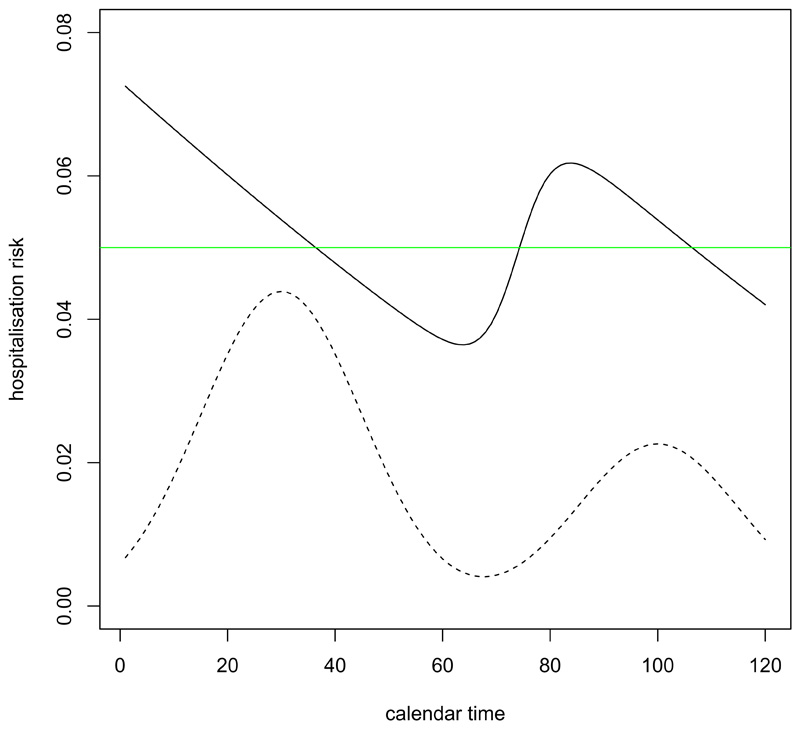
Hospitalisation risk conditional on positive test time (solid black line) when risk conditional on infection time is 0.05 (green line). Incidence of infection is shown (dotted line). Time from infection to positive test is assumed to have a gamma distribution with mean 4 and variance 8 for the ultimately hospitalised individuals and a gamma distribution with mean 7 and variance 14 for the ultimately non-hospitalised individuals.

Figure 2 shows how the hospitalisation risk conditional on positive test time varies according to positive test time in a scenario where the incidence of infection first rises then falls, then rises and falls again. Here, the hospitalisation risk conditional on infection time is 5% irrespective of the infection time, and the mean time from infection to positive test is shorter in individuals who are ultimately hospitalised than in those who will not.

## 4 Hospitalisation risk conditional on test time

As we have seen, conditioning on the positive test time changes the distribution of the delay in circumstances where the delay is independent of the time of infection. If hospitalisation is more common in individuals with shorter delays than in those with longer delays, i.e. *P*(*H* = 1 | *I* = *t*, *L* = *l*) is a decreasing function of *l*, then conditioning on the positive test time might be expected also to change the probability of hospitalisation. The effect of conditioning on *T* = *t* will depend on whether the incidence of infection is rising or falling. If it is rising, we might expect the proportion of hospitalisations to be increased, because short delays are over-represented. Conversely, if the incidence is falling, we might expect the proportion of hospitalisations to be decreased. We now confirm mathematically that this is true when the risk of hospitalisation either does not depend on the time of infection or changes little over the course of all but the longest delays.

Suppose that almost all delays are at most l* for some constant l* (i.e. *P*(*L* > l*) ≈ 0). Also, assume that

*P*(*H* = 1 | *I* = *t* – *l*, *L* = *l*) ≈ *P*(*H* = 1 | *I* = *t*, *L* = *l*) for all 0 < *l* ≤ l*. The risk of hospitalisation conditional on time of infection can then be written as

(3) $$\begin{array}{l} P\left(H=1|I=t\right)=\int_{0}^{\infty }P\left(H=1|I=t,L=l\right)f_{L}\left(l\right)dl\\ \;\approx \int_{0}^{l*}P\left(H=1|I=t,L=l\right)f_{L}\left(l\right)dl. \end{array}$$

Likewise, the risk of hospitalisation conditional on positive test time is

(4) $$\begin{array}{l} P\left(H=1|T=t\right)=\int_{0}^{\infty }P\left(H=1|I=t-l,L=l\right)f_{L}\left(l|T=t\right)dl\\ \;\approx \int_{0}^{l*}P\left(H=1|I=t-l,L=l\right)f_{L}\left(l|T=t\right)dl.\\ \;\approx \int_{0}^{l*}P\left(H=1|I=t,L=l\right)f_{L}\left(l|T=t\right)dl. \end{array}$$

Expressions (3) and (4) are both weighted averages of *P*(*H* = 1 | *I* = *t*, *L* = *l*). In expression (3) the weighting function is *f_L_*(*l*); in (4) it is *f_L_*(*l* | *T* = *t*). If the incidence of infection is increasing over the period [*t* – l*, *t*], then, as explained in Section 3, conditioning on *T* = *t* shifts probability mass from larger values of *L* to smaller values. Hence, the weighted average in expression (4) gives more weight to small values of *l* (and less weight to large values of *l*) than does the weighted average in expression (3). This, combined with our assumption that *P*(*H* = 1 | *I* = *t*, *L* = *l*) is a decreasing function of *l*, implies that (4) is greater than (3). That is, *P*(*H* = 1 | *T* = *t*) > *P*(*H* = 1 | *I* = *t*). On the other hand, if the incidence of infection is decreasing over the period [*t* – l*, *t*], then (as explained in Section 3) conditioning on *T* = *t* shifts probability mass from smaller values of *L* to larger values, with the result that *P*(*H* = 1 | *T* = *t*) < *P*(*H* = 1 | *I* = *t*).

*Example 1 continued*

Suppose *P*(*H* = 1 | *I* = *t*, *L* = 1) = 0.05 and *P*(*H* = 1 | *I* = *t*, *L* = 2) = 0.01. Then *P*(*H* = 1 | *I* = *t*) = (0.05 + 0.01)/2 = 0.03. If the incidence of infection is increasing, then of the 125 individuals who test positive on day *t*, the expected number who are hospitalised is 50 × 0.01 + 75 × 0.05 = 4.25, corresponding to a proportion of 4.25/125 = 0.034 (which is > 0.03). If, on the other hand, the incidence of infection is decreasing, then of the 125 individuals who test positive on day *t*, the expected number who are hospitalised is 75 × 0.01 + 50 × 0.05 = 3.25, corresponding to a proportion of 0.026 (which is < 0.03). The ratio of these two proportions is 0.034/0.026 = 1.31, and so the hospitalisation risk conditional on time of positive test would differ by 31% between a period of epidemic growth and a period of epidemic decline.

## 5 Hospitalisation risk conditional on infection time plus random delay

Suppose now that we had a different proxy of infection time, which, unlike positive test time, were not associated with the hospitalisation outcome. If we conditioned on this proxy, we might achieve the goal of approximately adjusting for time of infection without creating a measure of hospitalisation risk that depends on the trajectory of the infection incidence. We now describe such a proxy.

Suppose, hypothetically, that each individual who becomes infected at time *I* = *i* and tests positive at time *T* = *t* is randomly assigned a time variable *T*^0^ sampled from the conditional distribution of *T* given *I* = *i* and *H* = 0, i.e. the distribution of positive test time in those who are infected at the same time and who are not ultimately hospitalised. This time *T*^0^ will be our proxy of infection time. By construction, it is not associated with the hospitalisation outcome *H*.

We cannot actually carry out this assignment in practice, because we do not observe *I*. However, under the following working assumption, we shall still be able to estimate *P*(*H* = 1 | *T*^0^ = *t*).

### Assumption 1

$$f_{T}\left(t\;|\;I,H=1\right)=f_{T}\left(t+c|I,H=0\right),$$

*where c is some known constant.*

Assumption 1 means that the distribution of time from infection to positive test (conditional on time of infection) in ultimately hospitalised individuals equals the corresponding distribution in ultimately non-hospitalised individuals shifted by *c* days. So, the mean time from infection to test in the ultimately hospitalised is *c* days less than the mean in the ultimately non-hospitalised. We might expect that *c* > 0.

If Assumption 1 holds, then (see Appendix for proof)

(5) $$P\left(H=1\;|\;T^{0}=t\right)=P\left(H=1\;|\;T+cH=t\right).$$

It follows from equation (5) that *P*(*H* = 1 | *T*^0^ = *t*) can be consistently estimated simply by creating the new variable T* = *T* + *cH* (which equals *T* for ultimately non-hospitalised cases and *T* + *c* for ultimately hospitalised cases) and calculating the proportion who have *H* = 1 among those sampled individuals with T* = *t* (or, if time is continuous, T* ≈ *t*).

The hospitalisation risk conditional on *T*^0^, i.e. *P*(*H* = 1 | *T*^0^ = *t*), has the desirable property that *P*(*H* = 1 | *T*^0^ = *t*) = *P*(*H* = 1 | *I* = *t*) if *P*(*H* = 1 | *I* = *t*) does not depend on *t*. More generally, if *P*(*H* = 1 | *I* = *t*) ≈ *P*(*H* = 1 | *I* = *t* + *l*) for all 0 < *l* ≤ l*, then *P*(*H* = 1 | *T*^0^ = *t*) ≈ *P*(*H* = 1 | *I* = *t*). That is, when the risk conditional on *I* is constant or changes only slowly over time, conditioning on *T*^0^ yields almost the same risk as conditioning on *I*.

In practice, it is unlikely that we shall know the true value of *c*. However, one may be able to specify a range of plausible values for it and then investigate how sensitive the estimate of *P*(*H* = 1 | *T*^0^) is to this value. We shall illustrate this approach in Section 8.

Assumption 1 states that the distribution of delay in hospitalised individuals equals the distribution of delay in non-hospitalised individuals shifted by some number (*c*) of days. This assumption may well be false. In particular, it implies that the minimum delay in non-hospitalised cases cannot be less than *c*. So, in Section 6 we shall investigate the extent to which *P*(*H* = 1 | T*) differs from *P*(*H* = 1 | *T*^0^) when one delay distribution is not a shifted version of the other but we set *c* to be equal to *E*(*L* | *I*, *H* = 1) – *E*(*L* | *I*, *H* = 0), i.e. the difference between the mean of the two delay distributions.

## 6 Investigation of proposed method

**Figure 3:**
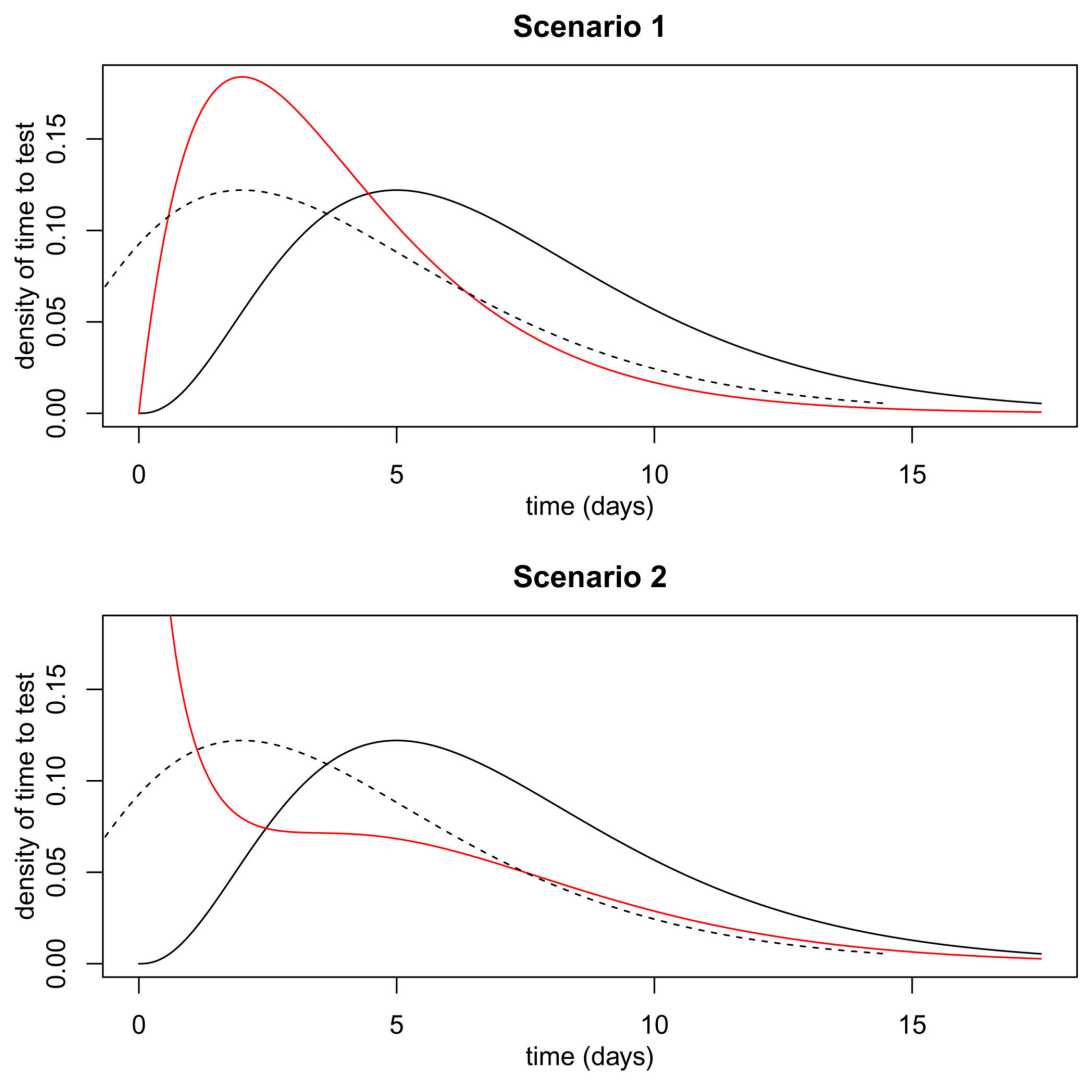
Distributions of time from infection to positive test. Solid black line is distribution for ultimately non-hospitalised individuals. Dotted line is same distribution shifted by three days. Red line is distribution for ultimately hospitalised individuals.

Suppose the incidence of infection at time *t* is proportional to exp(*λt*). If *λ* > 0, then *d* = log(2)/*λ* is the doubling time; if *λ* < 0, then −*d* is the halving time. Suppose that *P*(*H* = 1 | *I* = *t*), the risk of hospitalisation for an individual who is infected at time *t* is *r* = 0.05 and does not depend on the time of infection. Assume that the delay in the non-hospitalised cases has a gamma distribution with mean 7 and variance 14 (i.e. shape *α* = 3.5 and rate *β* = 0.5). We consider two scenarios for the distribution of delay in the hospitalised cases. In Scenario 1, it is a gamma distribution with mean 4 and variance 8 (shape *α*_1_ = 2 and rate *β* = 0.5). In Scenario 2, it is an equal mixture of a gamma distribution with mean 7 and variance 14 and a gamma distribution with mean 1 and variance 2 (shape *α*_2_ = 0.5 and rate *β* = 0.5). So, in both scenarios the difference between the mean delay in the hospitalised and non-hospitalised is three days. Figure 3 shows, for each scenario, the distributions of delay in the non-hospitalised (black) and hospitalised (red) cases. The dotted line shows the distribution in the non-hospitalised cases shifted by three days.

The number of non-hospitalised individuals who test positive at time *t* is proportional to

$$\left(1-r\right)\int_{-\infty }^{t}\exp\left(\text{λ}u\right)\frac{\beta^{\alpha }}{\Gamma \left(\alpha \right)}{\left(t-u\right)}^{\alpha -1}\exp\left\{-\beta \left(t-u\right)\right\}du=\left(1-r\right)\exp\left(\text{λ}t\right)\frac{\beta^{\alpha }}{{\left(\beta +\text{λ}\right)}^{\alpha }}.$$

Similarly, the number of hospitalised individuals who test positive at time *t* is proportional to

$$r\exp\left(\lambda t\right)\frac{\beta^{\alpha_{1}}}{{\left(\beta +\lambda \right)}^{\alpha_{1}}}$$

in Scenario 1 and is

$$r\exp\left(\lambda t\right)\times \frac{1}{2}\left\{\frac{\beta^{\alpha }}{{\left(\beta +\lambda \right)}^{\alpha }}+\frac{\beta^{\alpha_{2}}}{{\left(\beta +\lambda \right)}^{\alpha_{2}}}\right\}$$

in Scenario 2.

So, the risk when we condition on time of positive test *T* is

(6) $$P\left(H=1|T=1\right)=\frac{r\frac{\beta^{\alpha_{1}}}{{\left(\beta +\lambda \right)}^{\alpha_{1}}}}{\left(1-r\right)\frac{\beta^{\alpha }}{{\left(\beta +\lambda \right)}^{\alpha }}+r\frac{\beta^{\alpha_{1}}}{{\left(\beta +\lambda \right)}^{\alpha_{1}}}}$$

in Scenario 1 and is

(7) $$\begin{array}{l} P\left(H=1\;|\;T=t\right)=\frac{\frac{r}{2}\left\{\frac{\beta^{\alpha }}{{\left(\beta +\text{λ}\right)}^{\alpha }}+\frac{\beta^{\alpha_{2}}}{{\left(\beta +\text{λ}\right)}^{\alpha_{2}}}\right\}}{\left(1-r\right)\frac{\beta^{\alpha }}{{\left(\beta +\text{λ}\right)}^{\alpha }}+\frac{r}{2}\left\{\frac{\beta^{\alpha }}{{\left(\beta +\text{λ}\right)}^{\alpha }}+\frac{\beta^{\alpha_{2}}}{{\left(\beta +\text{λ}\right)}^{\alpha_{2}}}\right\}}\\ \;=\frac{r\left\{\frac{\beta^{\alpha }}{{\left(\beta +\text{λ}\right)}^{\alpha }}+\frac{\beta^{\alpha_{2}}}{{\left(\beta +\text{λ}\right)}^{\alpha_{2}}}\right\}}{\left(2-r\right)\frac{\beta^{\alpha }}{{\left(\beta +\text{λ}\right)}^{\alpha }}+r\frac{\beta^{\alpha_{2}}}{{\left(\beta +\text{λ}\right)}^{\alpha_{2}}}} \end{array}$$

in Scenario 2.

If we condition on T* = *T* + 3*H*, the risk is

(8) $$\begin{array}{l} P\left(H=1\;|\;T*=t\right)=\frac{r\exp\left\{\text{λ}\left(t-3\right)\right\}\frac{\beta^{\alpha_{1}}}{{\left(\beta +\text{λ}\right)}^{\alpha_{1}}}}{\left(1-r\right)\exp\left(\text{λ}t\right)\frac{\beta^{\alpha_{1}}}{{\left(\beta +\text{λ}\right)}^{\alpha }}+r\exp\left\{\text{λ}\left(t-3\right)\right\}\frac{\beta^{\alpha_{1}}}{{\left(\beta +\text{λ}\right)}^{\alpha_{1}}}}\\ \;=\frac{r\frac{\beta^{\alpha_{1}}}{{\left(\beta +\text{λ}\right)}^{\alpha_{1}}}}{\left(1-r\right)\exp\left(3\text{λ}\right)\frac{\beta^{\alpha }}{{\left(\beta +\text{λ}\right)}^{\alpha }}+r\frac{\beta^{\alpha_{1}}}{{\left(\beta +\text{λ}\right)}^{\alpha_{1}}}} \end{array}$$

in Scenario 1, and is

(9) $$\begin{array}{l} P\left(H=1\;|\;T*=t\right)=\frac{\frac{r}{2}\exp\left\{\lambda \left(t-3\right)\right\}\left\{\frac{\beta^{\alpha }}{{\left(\beta +\lambda \right)}^{\alpha }}+\frac{\beta^{\alpha_{2}}}{{\left(\beta +\lambda \right)}^{\alpha_{2}}}\right\}}{\left(1-r\right)\exp\left(\lambda t\right)\frac{\beta^{\alpha }}{{\left(\beta +\lambda \right)}^{\alpha }}+\frac{r}{2}\exp\left\{\lambda \left(t-3\right)\right\}\left\{\frac{\beta^{\alpha }}{{\left(\beta +\lambda \right)}^{\alpha }}+\frac{\beta^{\alpha_{2}}}{{\left(\beta +\lambda \right)}^{\alpha_{2}}}\right\}}\\ \;=\frac{r\left\{\frac{\beta^{\alpha }}{{\left(\beta +\lambda \right)}^{\alpha }}+\frac{\beta^{\alpha_{2}}}{{\left(\beta +\lambda \right)}^{\alpha_{2}}}\right\}}{\left\{\left(2-2r\right)\exp\left(3\lambda \right)+r\right\}\frac{\beta^{\alpha }}{{\left(\beta +\lambda \right)}^{\alpha }}+r\frac{\beta^{\alpha_{2}}}{{\left(\beta +\lambda \right)}^{\alpha_{2}}}} \end{array}$$

in Scenario 2.

Table 1 shows the results of applying equations (6)–(9) in Scenarios 1 and 2, when *d* = 4 and when *d* = 10. It also shows the results when *d* = −4 or *d* = −10, meaning that the incidence is falling with a halving time is 4 or 10 days. We see that the risks conditional on *T* are indeed different from the risk conditional on time of infection *I*, i.e. *r* = 0.05. The proposed method produces conditional risks *P*(*H* = 1 | T*) that are close to *r*.

Finally, Table 2 shows the risk ratios that Table 1 implies when comparing two variants both of which have the same risk *r* = 0.05, but one of which has a doubling time of 4 (respectively, 10) days and the other has a halving time of 4 (respectively, 10) days. The risk ratio conditional on time of infection is *r*/*r* = 1. The risk ratios conditional on *T* vary from 1.5 to 2.8. When we instead condition on T*, the conditional risk ratios vary from 0.95 to 1.04, i.e. they are much closer to 1.

## 7 Practical implementation when comparing risks of two variants

In practice, when comparing the risks of hospitalisation (or other post-infection outcome, like death) associated with two variants, researchers may adjust not only for positive test time but also for variables like age and ethnicity. In this section, we detail how researchers can apply our proposed sensitivity analysis in this situation. We shall begin by assuming that the researchers are using logistic regression (as did, e.g., [15, 16, 22]), and then go on to address the slightly more complicated situation where there is administrative censoring of the outcome and the researchers are using Cox regression or parametric survival regression (as did, e.g., [14, 17, 18, 19, 20, 21]).

Let *V* be a binary indicator for variant and *U* denote a vector of other variables that are fixed at the time of infection, e.g. age and ethnicity. Suppose the researchers have specified a particular logistic regression model, with *H*, the indicator of hospitalisation, as the outcome and with *V*, *U* and *T* (or functions of *T*, e.g. calendar week of positive test) as the covariates, and suppose they have fitted this model to all the individuals whose positive test time *T* lies in some chosen interval of calendar time [*τ*_1_, *τ*_2_]. The coefficient of *V* in this model represents the log odds of hospitalisation for variant adjusted for *U* and positive test time *T*.

To apply our method, assume that *f_T_*(*t* | *I*, *X*, *H* = 1) = *f_T_*(*t* + *c* | *I*, *X*, *H* = 0), where *X* = (*V*, *U*). This is like Assumption 1 but conditional on *X*, and it implies

(10) $$P\left(H=1\;|\;T^{0}=t,X\right)=P\left(H=1\;|\;T+cH=t,X\right)$$

(see Appendix for proof). Choose a range of plausible values for *c*. In Section 8 we illustrate how one might choose this range. Then, for any given value in this range (e.g. *c* = 1 day or *c* = 2 days), calculate T* = *T* + *cH* for each individual who has tested positive. Identify the set of individuals whose T* lies in the interval [*τ*_1_, *τ*_2_]. Note that this set differs from the set whose *T* lies in [*τ*_1_, *τ*_2_], because (assuming that *c* > 0) it excludes hospitalised individuals with *τ*_2_ – *c* < *T* ≤ *τ*_2_ and includes hospitalised individuals with *≤*_1_ – *c* ≤ *T* < *τ*_1_.^1^ Fit the same logistic regression model used earlier to this set of individuals, replacing the covariate *T* (or functions of *T*) in the model with a covariate T* (or functions of T*). The coefficient of V in this model is the log odds of hospitalisation for variant adjusted for *U* and T*. Equation (10) implies that this is also the log odds adjusted for *U* and *T*^0^, when the true value of *c* is used. The Supplementary Materials contain a simulation study that demonstrate the use of this method.

So far, we have assumed that the binary outcome *H* is observed for everyone who tests positive and that logistic regression has been used. In practice, there may be administrative censoring. This would occur if some of the sampled individuals test positive less than 14 days before time *τ*_2_, had not yet been hospitalised by time *τ*_2_, and no data were available on hospitalisations after time *τ*_2_. In this situation, researchers may use the *time* from positive test to hospitalisation as the outcome (rather than the binary indicator of hospitalisation within 14 days), right-censoring this time at 14 days, and fit a Cox regression model (or a parametric survival model) of this time-to-event outcome on *V*, *U* and *T* to all the individuals whose *T* lies in the interval [*τ*_1_, *τ*_2_]. When the binary outcome is rare and is fully observed, the hazard ratios (HRs) estimated from this Cox regression would be approximately equal to the odds ratio estimates from logistic regression [24].

Our proposed sensitivity analysis involves fitting exactly the same Cox regression (or parametric survival) model that the researchers have used, with exactly the same time-to-event outcome. The only difference is that: 1) the covariate *T* in this model is replaced by T*; and 2) this model is fitted to all the individuals whose T* lies in [*τ*_1_, *τ*_2_]. A difficulty arises because T* is calculated from *T* and *H*, the last of which is unobserved for the censored individuals. To avoid compromising the simplicity of the proposed method, we suggest assuming that individuals whose hospitalisation status is unknown due to this administrative censoring have *H* = 0 for the purpose of calculating T* (and so setting T* = *T*). Provided that hospitalisation within 14 days is uncommon, this assumption will be true for the great majority of censored individuals.

**Figure 4:**
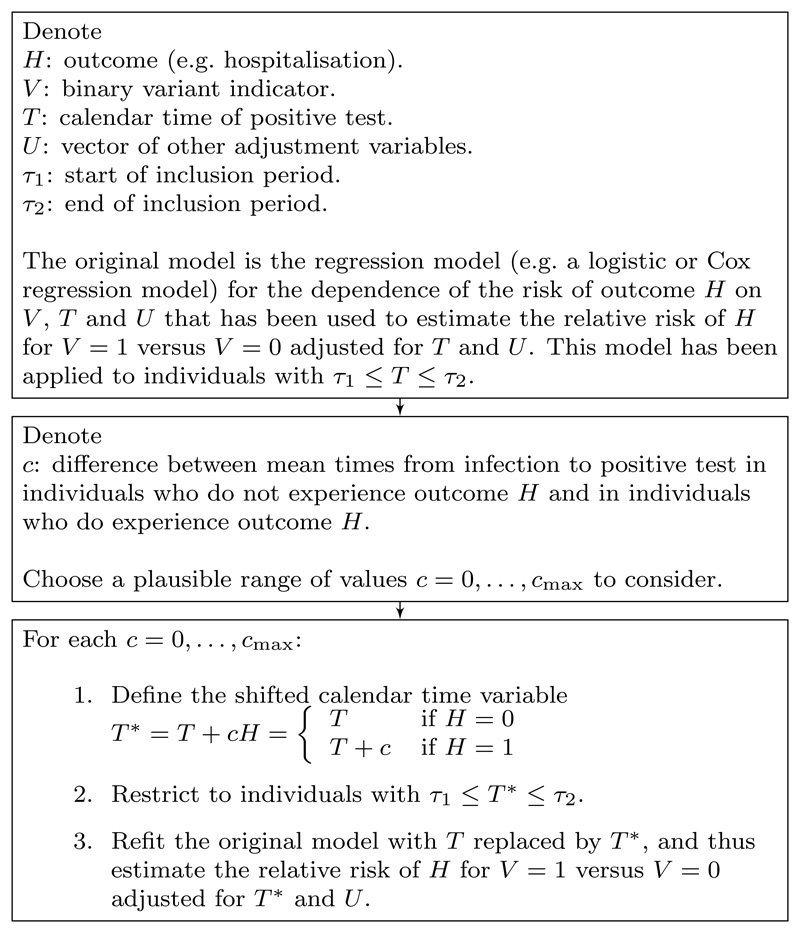
Summary of implementation of proposed sensitivity analysis.

Figure 4 summarises this procedure for carrying out the sensitivity analysis.

## 8 Application to COVID-19 hospitalisation and mortality data

### 8.1 Hazard ratio conditional on *T* and *T*^0^

Based on record linkage of routine healthcare data in England, Nyberg et al. (2021) recently reported a higher risk of hospital admission and mortality in COVID-19 cases infected with the Alpha variant (B.1.1.7) than in cases infected with pre-existing non-Alpha variants [18]. The study included data on 839,278 cases who had their first positive tests between 23 November 2020 and 31 January 2021 and whose positive test sample had been assessed for S gene target failure (SGTF). SGTF was used as a proxy test for the Alpha variant, and had positive and negative predictive values > 90% during the study period [25]. As determined by SGTF, 592,409 cases had Alpha and 246,869 cases had non-Alpha variants. Using stratified Cox regression, Nyberg et al. estimated that, after adjustment for date of positive test and other covariates, the HR of hospital admission within 14 days of positive test was 1.52 (95% CI 1.47–1.57) for Alpha versus non-Alpha cases. Full details on the dataset, including inclusion criteria, variant classification, outcome definitions, and adjustment methods, are available in the recent publication [18].

The Alpha variant was first detected in England in November 2020. During the study period the prevalence of the Alpha variant among newly test-positive cases, as determined by SGTF, increased from 16% in the week commencing 23 November 2020 to 94% in the week commencing 25 January 2021, and the prevalence of the pre-existing variants decreased accordingly [25]. During this period, the number of cases of the Alpha variant had a considerably higher growth rate than the number of cases of non-Alpha variants [26]. So, the aforementioned HR of 1.52 conditional on the positive test time *T* would be expected to be greater than the corresponding HR conditional on the infection time *I*.

We reanalysed the study dataset to estimate the HRs conditional on *T*^0^ for different choices of *c*. Columns 2 and 3 of Table 3 show the adjusted HR (and associated 95% CI) of hospital admission for Alpha versus non-Alpha from stratified Cox regression models fitted after adding *c* days to the date of first positive test for those cases who were ultimately hospitalised. The HR when *c* = 0 is that in the original publication [18]. As expected, there was a trend of decreasing HR with increasing *c*. The HR was greater than one and statistically significant when *c* was between 1 and 5 days.

### 8.2 Plausible upper bound for *c*

We now consider what might be a plausible upper bound for *c*. The cases used in the analysis of Nyberg et al. all tested positive through the pillar-two national SARS-CoV-2 testing programme. Pillar-two is a broad mass community testing programme of individuals seeking testing due to symptoms or contact tracing efforts, and 85.5% of the included cases reported COVID-19 symptoms at the time of their positive test (88.5% in hospitalised cases and 85.4% in non-hospitalised cases). The mean time from symptom onset to positive test was 2.65 days (standard deviation 2.13) in the symptomatic hospitalised cases, and 2.51 days (standard deviation 2.12) in the symptomatic non-hospitalised cases. These percentages and means were similar in both Alpha and non-Alpha cases (data not shown). These figures indicate no obvious association between hospitalisation and time from symptom onset to test. Thus, we might reasonably assume that the mean time is 2.5 days in both hospitalised and non-hospitalised cases.

It has been estimated that the mean incubation time (i.e. time from infection to symptom onset) in cases who eventually experience symptoms is 5.74 days [27]. Since most of the cases in the data set were not (ultimately) hospitalised, this can be regarded as the mean incubation time in non-hospitalised cases who eventually experience symptoms. We assume that the mean incubation time in hospitalised cases (all of whom must have been symptomatic or pre-symptomatic at time of positive test) is less than or equal to the mean time in non-hospitalised cases who eventually have symptoms, but is not less than two days.

We shall assume that the mean time from infection to positive test in the 14.6% of (ultimately) non-hospitalised cases who were asymptomatic at time of positive test is not more than 12 days. This choice of 12 days is somewhat arbitrary, but does not seem unreasonable given that some of these cases will have been pre-symptomatic at time of positive test. It now follows that the mean time from infection to positive test in the non-hospitalised cases is not more than 8.8 days (i.e. mean incubation 5.74 days plus mean time from symptoms to test 2.5 days for the 85.4% of symptomatic cases, and 12 days for the 14.6% of asymptomatic cases). Assuming that the mean time from infection to positive test in the hospitalised cases is at least 4.5 days (i.e. mean incubation 2 days plus mean time from symptoms to test 2.5 days), it follows that a plausible upper bound for *c* is 4.3 days (i.e. 8.8 – 4.5).

### 8.3 Analysis of symptomatic cases

The analysis described above includes a proportion (14.5%) of cases who were asymptomatic prior to their positive test. We now consider a modified analysis that focuses on symptomatic cases. It is the same as the analysis above, except that we now exclude cases who were both asymptomatic prior to positive test and not (ultimately) hospitalised.

4.3% (36,233/839,278) of the cases were hospitalised. After excluding the 117,494 non-hospitalised cases who were asymptomatic at time of positive test, 5.0% (36,233/721,784) of the remaining cases were hospitalised. Columns 4 and 5 of Table 3 show the results from analysing this subgroup consisting of symptomatic and/or hospitalised pillar-two cases (henceforth referred to as ‘symptomatic cases’). Consistent with the results using the full dataset, the HR point estimates were ≥ 1.10 when *c* ≤ 4 but was lower when *c* = 5.

Using the same logic as described above, the mean time from infection to positive test in the symptomatic non-hospitalised cases is not more than 8.2 days (i.e. mean incubation 5.74 days plus mean time from symptoms to test 2.5 days). Assuming again that the mean time from infection to positive test in the hospitalised cases is at least 4.5 days, a plausible upper bound for *c* is 3.7 days (i.e. 8.2 – 4.5).

### 8.4 Mortality

Using the same stratified Cox regression approach, Nyberg et al. (2021) also considered mortality as a secondary outcome, and reported an adjusted HR of death within 28 days of 1.59 (95% CI 1.44–1.74) for Alpha versus non-Alpha. Columns 6–9 of Table 3 show the original (*c* = 0) and adjusted HR of this outcome, after adding *c* days to the date of first positive test for those cases who ultimately died within 28 days of testing positive. There was a trend towards lower HR estimates with increasing *c*. The HRs were statistically significantly greater than 1 for *c* ≤ 4 when using all cases, and for *c* ≤ 3 when restricting to symptomatic cases, but non-significant for higher *c*.

## 9 Discussion

In this article we have highlighted the difference between the risk of a binary post-infection outcome (which, in this paper, is hospitalisation) conditional on the time of infection *I* and the corresponding risk conditional on the time of positive test *T*, and noted that the latter is a function of the trajectory of incidence of infection over calendar time. One way to interpret this difference is as a bias: if the goal is to estimate the risk conditional on infection time and if this risk differs from the risk conditional on the positive test time, then an (asymptotically) unbiased estimator of the latter risk will be an (asymptotically) biased estimator of the former risk. One might call this ‘epidemic phase bias’, since its direction and magnitude depend on whether the incidence of infection is falling or rising, and how quickly. This ‘bias’ may affect the results from a number of studies, e.g. [11, 12, 13, 14, 15, 16, 17, 18, 19, 20, 21, 22].

We have proposed a simple, easily implemented sensitivity analysis. This involves a third risk: that conditional on *T*^0^, the infection time plus a random time that is independent of the outcome. This third risk equals the risk conditional on infection time when the latter does not change over time (i.e. as a function of infection time), and is approximately equal to it when the risk conditional on infection time changes slowly over time. More generally, the two risks differ, but both have the advantage of not depending on the trajectory of incidence of infection.

As with other sensitivity analysis approaches, e.g. for addressing unmeasured confounding ([28]) and missing data ([29, 30]), ours does not yield a single estimate of the risk. It does, however, provide an indication of how sensitive the estimated risk is to the epidemic phase. If the incidence of infection is constant over calendar time, the estimated risk will not change as *c* is varied; if incidence is changing rapidly, the estimate will be very sensitive to the choice of *c*. When a risk ratio comparing two variants is of interest, sensitivity will be least when the variant-specific incidences of infection are both following the same trajectory (constant, increasing at the same exponential rate, or decreasing at the same rate), and will be greatest when one incidence is increasing rapidly and the other is decreasing rapidly.

The proposed method is likely to be most useful when a range of plausible values can be specified for *c*, the difference between the mean time from infection to positive test in the cases who experience the outcome and the corresponding mean time in those who do not experience the event. For our re-analysis of the healthcare data from England (Section 8) we suggested that the difference between these mean times is unlikely to be greater than 4.3 days when all cases are included. We found that the estimated hazard ratio (Alpha versus non-Alpha variant) of hospital admission within 14 days declined from 1.52 (when *c* = 0) to 1.41, 1.31, 1.21 or 1.13, respectively, when *c* = 1, 2, 3 or 4, although all HR estimates were statistically significantly greater than 1. When we restricted the analysis to symptomatic cases, we argued that a plausible upper bound for *c* is 3.7 days. The hazard ratio using these cases declined from 1.49 (when *c* = 0) to 1.37, 1.28, 1.19 or 1.10, respectively, when *c* = 1, 2, 3 or 4, and was still significant. The results were similar for the mortality outcome. Hence, although this sensitivity analysis does not refute the reported association between the Alpha variant and risk of hospital admission or mortality, the range of plausible values of *c* includes the possibility that differences in epidemic phase might have caused overestimation to some extent of the strength of association.

The true value of *c* is uncertain, however, and to establish the precise impact of the epidemic phase ‘bias’, further research is needed to estimate the difference, between ultimately hospitalised and non-hospitalised (or ultimately deceased and non-deceased) cases, in their mean time from infection to onset of symptoms, and their mean time from symptom onset to positive test. Of these, the difference in time from infection to symptom onset (i.e. incubation time) is the more uncertain. Estimates are available (e.g. [27]) for the mean incubation time in the population of all cases who experience symptoms. Since hospitalisation is an uncommon outcome, this mean should approximately equal the mean incubation time in ultimately non-hospitalised cases. Harder to estimate is the mean incubation time in cases who are ultimately hospitalised. For some cases it may be possible to establish retrospectively that they almost certainly became infected on a particular day, perhaps because they are known to have been exposed to an infected individual on that day and unlikely to have been exposed otherwise during the period around that day. If a sufficient number of such cases were identified, it ought to be possible to use them to estimate the mean incubation time in ultimately hospitalised and non-hospitalised cases separately, and hence the difference between these means. However, the numbers of cases required to do this with precision could be large, because hospitalisation is uncommon. There might also be a concern that these cases with known infection times were unrepresentative of all cases.

It would be straightforward in theory to allow *c* to depend on observed variables *X*. Simply assume that *f_T_*(*t* | *I*, *X*, *H* = 1) = *f_T_*{*t* + *c*(*X*) | *I*, *X*, *H* = 0), for some function *c*(*x*) of *x*, and redefine T* slightly as T* = *T* + *c*(*X*)*H*. One might also want to allow *c* to depend on the unknown infection time *I*. For example, if the mean time from infection to positive test in the non-hospitalised cases is getting smaller over the study period, then it is quite likely that the difference between the mean time in non-hospitalised cases and the mean time in hospitalised cases is also getting smaller. If it were necessary to allow *c* to depend on *I* over the study period, then a crude but practical way of doing this would be to specify *c* as a function of *T* and calculate T* as T* = *T* + *c*(*T*).

We have focused on an observed binary outcome *H*, but also briefly addressed right-censoring of this outcome. We proposed that T* be calculated as though the censored individuals did not experience the outcome (i.e. *H* = 0). This is a reasonable approximation when the outcome is rare and the proportion of censored individuals is small. For more common outcomes or when the extent of censoring is larger, it would be preferable to use a more sophisticated approach. More research is needed on this, but one possibility may be the following. Fit the Cox model to the original data. Estimate the baseline hazard. Use this estimated baseline hazard and the estimated hazard ratios from the Cox model to calculate the probability *p_i_* that a censored individual i has *H* = 1. Then create two copies of each censored individual *i*: one with *H* = 1, T* = *T* + *c* and weight *p_i_*; and one with *H* = 0, T* = *T* and weight 1 – *p_i_*. An obvious drawback of this method is that *p_i_* would be calculated from a model that implicitly assumed *c* = 0. A more refined version might begin by calculating T* as though the censored individuals all had *H* = 0, then using the resulting fitted Cox model and estimated baseline hazard to calculate *p_i_*.

We have assumed that all infections result in a positive test. This is obviously not true in reality. However, this issue affects all studies of risks of post-infection outcomes in samples of individuals who have tested positive, and is not specific to this article. There is not a problem if those individuals who test positive are representative of all infected individuals. Otherwise, the estimated risks must be interpreted as risks conditional on eventually testing positive.

We have focused on a setting where most delays are measured in days or a small number of weeks. Here, researchers may view time of positive test as a good proxy for time of infection. In a setting where the mean delay is much larger or where there is a long tail in the delay distribution, both the actual positive test time *T* and its shifted counterpart T* will typically be very different from the infection time *I*. In that setting, although adjustment for T* can still remove the dependence of the risk on the trajectory of incidence to which the risk adjusted for *T* is subject, neither of these adjusted risks should be seen as an approximation of the risk conditional on *I*.

Finally, if additional information is available on the incidence of infection with each variant over time, it may be possible to estimate the hospitalisation risk without using data on positive test times. This could be done using deconvolution techniques, such as those developed in the 1980’s and 1990’s for back-calculation in the context of the HIV/AIDS epidemic. There the purpose was to estimate the distribution of HIV infection times from the observed distribution of AIDS onset times and an assumed-known distribution of time from infection to AIDS onset. For example, Rosenberg and Gail (1991) described how to do this using software for Poisson regression with identity link function [31]. In the context of the present article, the purpose would be to estimate, for each variant, the distribution of time from infection to hospitalisation from the observed distribution of hospitalisation times and an assumed-known distribution of infection times. It may be possible to do this by applying, for example, an adaptation of the Poisson regression method of Rosenberg and Gail with an additional offset term for the total number of infections observed so far.

## Supplementary Material

Supplementary file

Appendix

## Figures and Tables

**Table 1:** Risks when adjusted for *T* and T*. If *d* = 4 or 10, the doubling time is 4 or 10 days. If *d* = –4 or –10, the halving time is 4 or 10 days.

Scenario	*d*	*T*	T*
1	4	0.0760	0.0466
1	10	0.0601	0.0494
1	–4	0.0270	0.0447
1	–10	0.0404	0.0493
2	4	0.0830	0.0511
2	10	0.0612	0.0503
2	–4	0.0326	0.0536
2	–10	0.0414	0.0504

**Table 2:** Risk ratios when adjusted for *T* and T*. One variant has doubling time *d* = 4 (or *d* = 10) days and the other has halving time *d* = 4 (or *d* = 10) days.

Scenario	*d*	*T*	T*
1	4	2.810	1.044
1	10	1.489	1.003
2	4	2.551	0.954
2	10	1.480	0.997

**Table 3:** Hazard ratios for the two outcomes hospital admission and death (and 95% confidence intervals) conditional on T* for COVID-19 cases with Alpha compared to non-Alpha variants, as determined based on S gene target failure. The assumed difference in mean number of days from infection to first positive test between individuals without the outcome and with the outcome is *C*. HRs were estimated using stratification for calendar week of positive test, age group, sex, ethnicity, index of multiple deprivation quintile, and region of residence (Public Health England Centres); and including strata-specific linear terms for exact date of positive test, exact age, and index of multiple deprivation rank; see [18].

Hospitalisation outcome					Death outcome			
	All COVID cases		Symptomatic cases		All COVID cases		Symptomatic cases	
*c*	HR	(95% CI)	HR	(95% CI)	HR	(95% CI)	HR	(95% CI)
0	1.52	(1.47–1.57)	1.49	(1.44–1.54)	1.59	(1.44–1.74)	1.42	(1.29–1.56)
1	1.41	(1.36–1.45)	1.37	(1.33–1.42)	1.43	(1.30–1.57)	1.32	(1.20–1.45)
2	1.31	(1.27–1.35)	1.28	(1.24–1.32)	1.33	(1.21–1.46)	1.23	(1.12–1.35)
3	1.21	(1.17–1.25)	1.19	(1.15–1.22)	1.23	(1.12–1.35)	1.14	(1.04–1.25)
4	1.13	(1.09–1.16)	1.10	(1.07–1.14)	1.15	(1.04–1.26)	1.07	(0.97–1.17)
5	1.04	(1.01–1.07)	1.02	(0.99–1.05)	1.06	(0.97–1.17)	1.00	(0.91–1.10)

CRP, C-reactive protein; AGP, α-1-acid glycoprotein.

## Data Availability

This analysis was based on routine healthcare data, which cannot be made available to others by the study authors. Requests to access these non-publicly available data are handled by the Public Health England Office for Data Release (https://www.gov.uk/government/publications/accessing-public-health-england-data/about-the-phe-odr-and-accessing-data).

## Footnotes

1 Alternatively, if data on individuals with *T* < *τ*_1_ are unavailable, one can instead identify the set of individuals whose *T** lies in the interval [*τ*_1_ + *c*; *τ*_2_].
