# Supplementary material for: Adjusting for time of infection or positive test when estimating the risk of a post-infection outcome in an epidemic
Source: Stat Methods Med Res. 2022 Jun 12;31(10):1942–58. doi: 10.1177/09622802221107105 (PMC7613654; doi:10.1177/09622802221107105)
Supplement: sj-pdf-1-smm-10.1177_09622802221107105 - Supplemental material for Adjusting for time of infection or positive test when estimating the risk of a post-infection outcome in an epidemic [file sj-pdf-1-smm-10.1177_09622802221107105.pdf]

Supplementary Material for "Adjusting for time of infection or positive test when estimating the risk of a post-infection outcome in an epidemic"

by Shaun R. Seaman, Tommy Nyberg, Christopher E. Overton, David J. Pascall, Anne M. Presanis and and Daniela De Angelis

Here we report a simulation study that demonstrates the use of the logistic regression method described in Section 7 of our article. R code for performing this simulation study is also included in the Supplementary Materials.

We estimate the odds ratio for variant $V = 1$ (compared to variant $V = 0$) adjusted for infection time I, positive test time T, and shifted positive test time T^*. For each of three scenarios (see below), 5000 data sets each consisting of 10000 infected individuals were generated using the following data-generating mechanism.

Each individual's infection time I was generated from a Uniform$(0, 119)$ distribution, meaning that the incidence of infection is constant over time over the period from time 0 to time 119. Times 0 and 119 correspond to the beginning of week 1 and the end of week 17, respectively.

The probability that an infection that occurred at time t was caused by variant 1 is

$$P(V = 1 \mid I = t) = \frac{\exp(-3.5 + 0.05t)}{1 + \exp(-3.5 + 0.05t)}$$

meaning that the proportion of infections that are due to variant 1 increases over time (see Figure 1). For each individual, data were generated on three variables $U = (U_1, U_2, U_3)$ with multivariate normal distribution

$$(U_1, U_2, U_3) \mid V, I \sim \text{Normal}_3(0_3, I_3).$$

The binary hospitalisation indicator H was then generated for each individual, with

$$P(H = 1 \mid U, V, I) = \frac{\exp(-4.4 + 1.5U_1 + U_2 + 0.5U_3 + 0.4V)}{1 + \exp(-4.4 + 1.5U_1 + U_2 + 0.5U_3 + 0.4V)}. \tag{1}$$

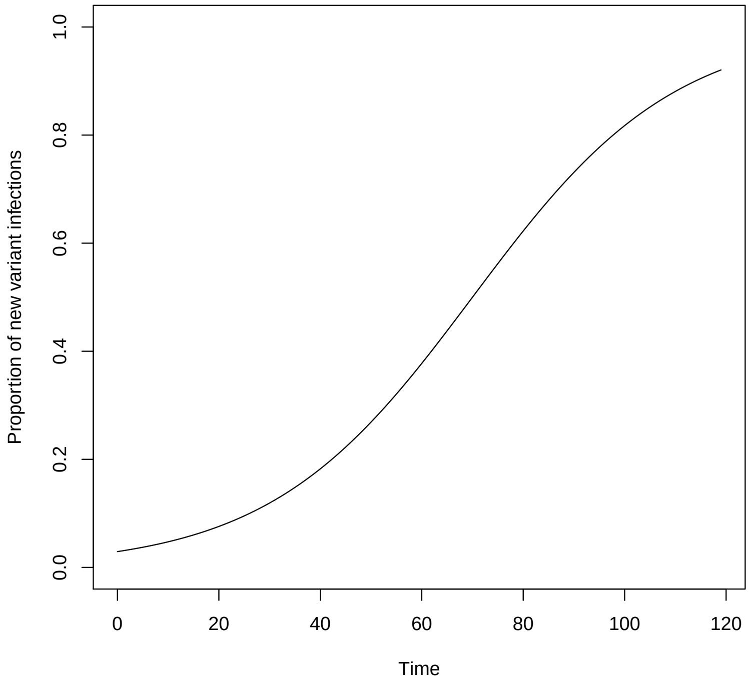

Figure 1: Proportion of infections caused by variant 1 as a function of infection time.

Hence, the risk of hospitalisation given variant, infection time and U does not depend on infection time, and the log odds ratio for variant adjusted for the infection time and U is 0.4. The marginal probabilities of hospitalisation for the two variants were $P(H = 1 \mid V = 0) = 0.45$ and $P(H = 1 \mid V = 1) = 0.61$.

The lag $L = T - I$ was assumed to be independent of V, U and I given H, and three scenarios were considered for the distribution of L given H:

Scenario A :

$$L \mid H = 1 \sim \text{Gamma}(2, 0.5)$$
$$L \mid H = 0 \sim \text{Gamma}(2, 0.5) + 3.$$

Scenario B :

$$L \mid H = 1 \sim \text{Gamma}(2, 0.5)$$
$$L \mid H = 0 \sim \text{Gamma}(3.5, 0.5)$$

Scenario C :

$$L \mid H = 1 \sim 0.5\ \text{Gamma}(0.5, 0.5) + 0.5\ \text{Gamma}(3.5, 1)$$
$$L \mid H = 0 \sim \text{Gamma}(3.5, 0.5)$$

Here, $\text{Gamma}(\alpha, \beta)$ denotes a Gamma distribution with shape α and rate β. Scenario A represents a situation where the assumption that $f_T(t \mid I, V, U, H = 1) = f_T(t + c \mid I, V, U, H = 0)$, is true, with $c = 3$, and Scenarios B and C were the scenarios used in Section 6 of our article. In all three scenarios, the mean lag for non-hospitalised cases is three days longer than the mean lag for hospitalised cases, and so we define $T^* = T + 3$.

Three logistic regression models were fitted to each data set:

$$\text{Model } I\text{:} \quad \text{logit } P(H = 1 \mid U, V, I) = \sum_{j=4}^{17} \alpha_{0j} 1_{\{I_{\text{week}}=j\}} + \alpha_{u1}U_1 + \alpha_{u2}U_2 + \alpha_{u3}U_3 + \alpha_v V$$

$$\text{Model } T\text{:} \quad \text{logit } P(H = 1 \mid U, V, T) = \sum_{j=4}^{17} \beta_{0j} 1_{\{T_{\text{week}}=j\}} + \beta_{u1}U_1 + \beta_{u2}U_2 + \beta_{u3}U_3 + \beta_v V$$

$$\text{Model } T^*\text{:} \quad \text{logit } P(H = 1 \mid U, V, T^*) = \sum_{j=4}^{17} \gamma_{0j} 1_{\{T^*_{\text{week}}=j\}} + \gamma_{u1}U_1 + \gamma_{u2}U_2 + \gamma_{u3}U_3 + \gamma_v V$$

where $1_{\{\}}$ is the indicator function and I_{week}, T_{week} and T^*_{week} are defined as follows:

$$I_{\text{week}} = 1 \text{ if } 0 \le I < 7;\ I_{\text{week}} = 2 \text{ if } 7 \le I < 14;\ \ldots;\ I_{\text{week}} = 17 \text{ if } 113 \le I < 119$$
$$T_{\text{week}} = 1 \text{ if } 0 \le T < 7;\ T_{\text{week}} = 2 \text{ if } 7 \le T < 14;\ \ldots;\ T_{\text{week}} = 17 \text{ if } 113 \le T < 119$$
$$T^*_{\text{week}} = 1 \text{ if } 0 \le T^* < 7;\ T^*_{\text{week}} = 2 \text{ if } 7 \le T^* < 14;\ \ldots;\ T^*_{\text{week}} = 17 \text{ if } 113 \le T^* < 119.$$

The parameters α_v, β_v and γ_v in Models I, T and T^* are the log odds ratio for variant adjusted for the U and, respectively, the infection time, the positive test time and the shifted positive test time.

Model I was fitted to the individuals with $4 \le I_{\text{week}} \le 17$. Model T was fitted to the individuals with $4 \le T_{\text{week}} \le 17$. Model T^* was fitted to the individuals with $4 \le T^*_{\text{week}} \le 17$.

		Scenario		
Model	Parameter	A	B	C
I	α_v	0.40	0.40	0.40
T	β_v	0.55	0.55	0.55
T^*	γ_v	0.40	0.40	0.40

Table 1: Mean estimates of the log odds ratios α_v, β_v and γ_v in the three logistic regression models.

Table 1 shows the mean (over the 5000 simulated datasets) of the estimates for α_v, β_v and γ_v. As expected, the mean estimates of α_v and γ_v both equal 0.40 and the mean estimate of β_v is greater than that (it is 0.55). A log odds ratio of 0.40 corresponds to an odds ratio of 1.49, while an log odds ratio of 0.55 corresponds to an odds ratio of 1.73, almost 50% higher. This illustrates the 'epidemic phase bias' and the removal of this 'bias' by adjusting for the shifted positive test time.
