# Supplementary material for: Adjusting for time of infection or positive test when estimating the risk of a post-infection outcome in an epidemic
Source: Stat Methods Med Res. Author manuscript; Available in PMC 2022 Oct 1. (PMC7613654; doi:10.1177/09622802221107105)
Supplement: Supplementary file 1 [file EMS145047-supplement-Appendix.pdf]

Appendix: Proof of equations (5) and (10)

Since equation (5) is a special case of equation (10), it suffices to prove that the latter is implied by the equation $f_T(t \mid I, X, H = 1) = f_T(t + c \mid I, X, H = 0)$.

The probability distribution function of the observed data (X, T, H) at (x, t, h) is

$$f_{X,T,H}(x, t, h) = \int f_I(i)\ f_{H|I}(h \mid i)\ f_{X|I,H}(x \mid i, h)\ f_{T|I,X,H}(t \mid i, x, h)\ di.$$

By definition, T^0 is sampled from

$$f_{T|I,X,H}(t \mid i, x, 0)$$

independently of T. So, the joint probability distribution function of (X, T^0, H) evalulated at (x, t, h) is

$$f_{X,T^0,H}(x, t, h) = \int f_I(i)\ f_{H|I}(h \mid i)\ f_{X|I,H}(x \mid i, h)\ f_{T|I,X,H}(t \mid i, x, 0)\ di. \tag{11}$$

Let $T^* = T + cH$. Then the joint probability distribution function of (X, T^*, H) evalulated at (i, t, h) is

$$f_{X,T^*,H}(x, t, h) = \int f_I(i)\ f_{H|I}(h \mid i)\ f_{X|I,H}(x \mid i, h)\ f_{T|I,X,H}(t - hc \mid i, x, h)\ di. \tag{12}$$

Assumption 1 can be written as

$$f_{T|I,X,H}(t - c \mid i, x, 1) = f_{T|I,X,H}(t \mid i, x, 0).$$

So, equation (12) becomes

$$f_{X,T^*,H}(x, t, h) = \int f_I(i)\ f_{H|I}(h \mid i)\ f_{X|I,H}(x \mid i, h)\ f_{T|I,X,H}(t \mid i, x, 0)\ di. \tag{13}$$

The right-hand sides of lines (13) and (11) are the same. So, (X, T^*, H) and (X, T^0, H) have the same joint distribution. Therefore, the conditional distribution of H given X and T^0 is the same as the conditional distribution of H given X and T^*.
